# Predictors of Remission in Graves’ Disease Patients Treated With Antithyroid Drugs: A Retrospective Study

**DOI:** 10.7759/cureus.77380

**Published:** 2025-01-13

**Authors:** Raghad S Alzahrani, Yazeed H Aljabri, Wasan S Alzahrani, Feras M Fatani, Loay J Alghamdi, Ali S Alsudais, Suhaib Radi

**Affiliations:** 1 College of Medicine, King Saud Bin Abdulaziz University for Health Sciences, Jeddah, SAU; 2 Endocrinology, King Abdullah International Medical Research Center, Jeddah, SAU; 3 Endocrinology, Ministry of the National Guard - Health Affairs, Jeddah, SAU

**Keywords:** antithyroid drugs, graves’ disease, hyperthyroidism, predictors, remission

## Abstract

Background and objectives: Graves' disease (GD) is an autoimmune disorder characterized by excessive stimulation of the thyroid gland, resulting in hyperthyroidism. Antithyroid drugs (ATDs) are commonly used for its treatment, but the predictors of remission and factors associated with a positive response to ATD therapy are not fully understood.

Methods: A retrospective study was conducted using medical records of 64 patients diagnosed with GD at a hospital in Saudi Arabia. Demographic characteristics, thyroid-related factors, ATD treatment details, and remission rates data were collected. Statistical analysis was performed using univariate association tests and multivariable logistic regression models.

Results: The majority of participants were females, with a median age of 38 years. Smoking history was reported in 28.9% of patients. Methimazole (MMI) was the predominant ATD used. The remission rate with ATD treatment was 62.5%. In terms of predictors of remission, 50% of female participants achieved remission compared to 94.4% of male participants (p < 0.01). The body mass index was 26.4 in the remitted group compared to 30.4 in those not achieving remission (p = 0.028). The maximum TSH level while on treatment exhibited significant association as the remitted group had higher maximum TSH levels (median 6.5) compared to the non-remitted group (median 1.2) (p = 0.010).

Conclusion: The study highlights the common use of MMI as the primary antithyroid drug and the positive remission outcomes in most patients. Male gender, lower BMI, and higher maximum TSH levels on treatment were predictors of remission. More research with larger samples and longer follow-up is needed.

## Introduction

The incidence of autoimmune diseases has significantly risen, with type 1 diabetes mellitus and autoimmune thyroid disease being the most prevalent conditions [1]. Graves' disease (GD) is an autoimmune disorder characterized by excessive thyroid gland stimulation due to circulating thyroid stimulating hormone (TSH) receptor autoantibodies, leading to hyperthyroidism. The disease mechanism involves autoimmune reactions by B and T cells, resulting in the production of immunoglobulin G (IgG) against the TSH receptor, which enlarges the thyroid and increases its function. Elevated levels of thyroid-stimulating hormone receptor antibodies (TRAbs) lead to hyperthyroidism and diffuse goiter [2]. The presence of thyroid receptor antibodies (TRAbs) is strongly associated with GD, detectable in up to 98% of patients [3]. It is one of the leading causes of hyperthyroidism, predominantly affecting women [2,4]. GD is characterized by symptoms such as anxiety, weight loss, goiter, and Graves' ophthalmopathy [2,5-7].

GD is the primary cause of hyperthyroidism in the United States [8], predominantly affecting women with a lifetime risk of 3% compared to 0.5% in men [4]. Although GD typically occurs between 30 and 50 years of age, it can manifest at any stage of life [4]. A recent study in the Asir region of Saudi Arabia reported primary hyperthyroidism accounting for 2.5% of overall thyroid dysfunction prevalence [9]. The risk factors for GD include infections, pregnancy, smoking, stress, family history, and other autoimmune disorders. Genetic factors also play a role in susceptibility to the disease. Furthermore, certain genes, such as HLA-DR3 and HLA-DR8, confer susceptibility to GD [10].

The diagnosis of GD involves clinical examination and tests to measure thyroid hormone levels and detect the presence of thyroid-stimulating hormone receptor antibodies [11]. Investigative tests are essential for confirming the diagnosis. In GD, there is typically an elevation in T3 and T4 levels accompanied by low TSH levels due to negative feedback inhibition caused by high T4 levels [12]. Imaging techniques such as iodine-123 scanning and thyroid ultrasound can aid in the diagnosis and evaluation of the disease [8,13].

The treatment of GD involves various approaches depending on the individual patient and disease severity. Antithyroid drugs (ATDs), such as methimazole (MMI) and propylthiouracil (PTU), are commonly used to inhibit hormone production and achieve a euthyroid state [14]. Beta-blockers may be prescribed to manage hyperthyroidism symptoms [15]. Radioactive iodine ablation and thyroidectomy are options for patients with small goiters or in cases where other treatments are not effective or suitable [15,16].

The remission rate of GD varies across different studies, typically ranging from 30% to 70% [17]. On average, about 50% of patients achieve remission after completing a course of ATDs. However, it is important to note that recurrence of the disease is possible, with most cases occurring after a period of four years of remission [18]. After 10 years of remission, recurrence rates can range from 30% to 40%, and after 15 years, only 10% to 15% of patients experience hyperthyroidism again [4]. Unfortunately, permanent remission is observed in only 27% of cases, with a ratio of one to three [17,19].

Several studies have explored potential factors associated with remission in GD. One study determined that the lack of Graves orbitopathy and possessing the CTLA-4 G/G genotype independently predict remission in GD patients [20]. Another study identified baseline TRAbs laboratory values as a prognostic indicator for remission in GD [21]. However, there is currently no local comprehensive study that thoroughly investigates predictors of GD remission specifically in patients undergoing ATD treatment.

The aim of this study is to investigate the use of ATDs in the treatment of GD, including assessing remission rates and identifying predictors of a positive response to antithyroid therapy. The study will be conducted at the National Guard Hospital in Jeddah, Saudi Arabia.

## Materials and methods

Study cohort

This study was conducted after receiving the Institutional Review Board approval number (IRB/1987/22) from King Abdullah International Medical Research Center in September 2022. We conducted a retrospective analysis of medical records from the main hospital of King Abdulaziz Medical City in Jeddah, Saudi Arabia. The study included 64 patients who were diagnosed with GD between January 2015 and January 2022. GD was confirmed if the patient had thyrotoxicosis along with one or more of the following: orbitopathy, positive TSH-receptor antibodies, and/or diffuse uptake on a thyroid scan. To be included in the study, patients must have received treatment with ATDs (MMI, carbimazole, or PTU) at some point during their disease course. Patients with a follow-up duration of less than one year or those who were lost to follow-up were excluded. We also excluded patients with uncertain laboratory and imaging results for GD, patients with thyrotoxicosis caused by factors other than GD, and patients who did not receive ATDs.

Data collection

We collected various characteristics of the participants, including age, body mass index (BMI), comorbidities, and smoking status, from the electronic (BESTCare, ezCaretech, USA) system. This information was gathered for patients treated between 2015 and 2022 using a data collection form. We also collected information on the signs and symptoms of GD, such as the history of goiter and orbitopathy, along with their severity. In addition, we documented the results of diagnostic laboratory tests before, during, and after treatment with ATDs, including thyroid-stimulating hormone (TSH), free T4, and free T3 levels. Details regarding the type of ATD, dosage, and duration of treatment were recorded, as well as information on remission or recurrence of the disease. Finally, we documented any side effects experienced by the patients, such as neutropenia, liver failure, and rash. Liver failure was defined as a three-fold increase in aspartate transaminase (AST) or alanine transaminase (ALT) concentrations, while neutropenia was defined as a neutrophil count below 1 x10^9^ /L.

Statistical analysis

We performed the data analysis using RStudio (R version 4.3.0, Posit PBC, USA). Categorical variables were presented as frequencies and percentages, while numerical variables were expressed as median and interquartile range (IQR). For inferential analysis, we conducted univariate association tests, including Fisher's exact test or Pearson's chi-square test for categorical variables, and Wilcoxon rank-sum test for numerical variables. Variables that showed statistical significance in the univariate analysis were further analyzed using multivariable logistic regression models to identify predictors of remission or relapse. The results of the regression analysis were reported as odds ratios (ORs) with corresponding 95% confidence intervals (CIs). Statistical significance was considered at a p-value of less than 0.05.

## Results

Demographic and thyroid-related characteristics

**Figure 1:**
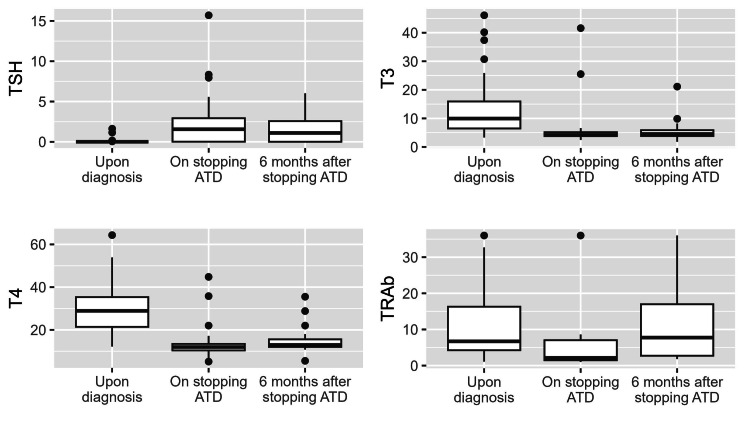
Boxplots depicting the concentrations of the selected laboratory parameters. The laboratory values of TSH upon diagnosis, at stopping ATD, and six months after stopping ATD were 0.01 (0.01-0.01), 1.56 (0.02-2.93), and 1.11 (1.01-2.57), respectively. The laboratory values of FT3 upon diagnosis, at stopping ATD, and six months after stopping ATD were 9.95 (6.48-15.93), 4.22 (3.80-5.17), and 4.56 (3.82-5.88). The laboratory values of FT4 upon diagnosis, at stopping ATD, and six months after stopping ATD were 28.90 (21.40-35.33), 11.97 (10.38-13.43), and 12.96 (12.01-15.56). The laboratory values of TRAb upon diagnosis, at stopping ATD, and six months after stopping ATD were 6.70 (4.25-16.30), 2.15 (1.48-7.03), and 7.75 (2.73-16.98). Abbreviations: ATD: anti-thyroid drugs; TSH: thyroid-stimulating hormone; TRAb: TSH-receptor antibodies; FT4: free T4; FT3: free T3. Reference ranges and units: TSH: 0.4-4 mIU/L; FT3: 1.6-4 pmol/L; FT4: 9-19 pmol/L; TRAb: < 2 IU/L

In Table 1, the demographic and baseline characteristics of the study population (N = 64) are presented. The majority of participants were females (71.9%), with a median age at diagnosis of 38.0 years (interquartile range (IQR): 30.0-51.3). The median duration of follow-up was 5.0 years (IQR: 3.0-6.0). Smoking history was reported in 28.9% of patients. The median BMI at diagnosis was 27.6 (IQR: 24.9-33.0). Goiter was observed in 66.0% of patients. Among those with available data, the median volume/weight of the thyroid gland was 31.2 grams (IQR: 29.1-51.5). Regarding thyroid lobe sizes, the median size of the right lobe was 5.1 cm (IQR: 4.4-5.8), and the median size of the left lobe was 4.8 cm (IQR: 4.3-5.4). Orbitopathy was present in 31.1% of patients. Regarding laboratory values, Figure 1 depicts the descriptive analyses of the participants’ laboratory parameters upon diagnosis, on stopping ATDs, and at six months after stopping ATDs.

**Table 1:** : Demographics and baseline characteristics Wilcoxon rank-sum test was used for numerical variables, and Fisher’s exact test or Pearson’s chi-squared test was used for categorical variables. A p-value <0.05 was considered statistically significant. *n (%) is calculated based on available records. Abbreviations: BMI: body mass index; ATD: anti-thyroid drugs; TSH: thyroid-stimulating hormone

Characteristic	Median (IQR); n (%)*
Gender	
Male	18 (28.1%)
Female	46 (71.9%)
Age at diagnosis in years	38.0 (30.0-51.3)
Duration of follow-up in years	5.0 (3.0-6.0)
Smoking history	11 (28.9%)
BMI in kg/m^2^	27.6 (24.9-33.0)
Presence of goiter	33 (66.0%)
Volume/weight of thyroid gland in grams	31.2 (29.1-51.5)
Size of the right lobe of the thyroid gland in cm	5.1 (4.4-5.8)
Size of the left lobe of the thyroid gland in cm	4.8 (4.3-5.4)
Presence of orbitopathy	14 (31.1%)
Degree of uptake on thyroid scan in %	6.0 (2.6-11.7)
Type of ATD	
Methimazole	63 (98.4%)
Propylthiouracil	1 (1.6%)
Average dose of medication in mg/day	10.0 (7.5-15.0)
Maximum TSH while on treatment in microU/mL	5.2 (1.7-10.6)

Characteristics of ATD

Table 1 outlines the characteristics of ATD treatment among the study participants (N = 64). MMI was the predominant ATD used, accounting for 98.4% of cases. The median average dose of MMI administered was 10.0 mg/day (IQR: 7.5-15.0). The degree of uptake on thyroid scan demonstrated a median value of 6.0% (IQR: 2.6-11.7). The maximum TSH level observed during treatment was 5.2 microU/mL (IQR: 1.7-10.6).

Rates of remission

Among the patients under study, 40 patients reported remission from ATDs, accounting for 62.5% of the study population (Table 2). ATDs have been stopped upon remission in 50.0% of patients, and the median duration of ATD use till remission was 14.5 months (IQR = 10.3-22.8), while the total duration of ATD use was 22.5 months. Among the 24 patients with persistent disease, we have records on 21 of them. Fifteen out of those 21 achieved remission with second-line therapies (11 with radioactive iodine and four with thyroidectomy) (Table 2).

**Table 2:** Characteristics of remission and relapse Wilcoxon rank-sum test was used for numerical variables, and Fisher’s exact test or Pearson’s chi-squared test was used for categorical variables. A p-value <0.05 was considered statistically significant. Abbreviations: ATD: anti-thyroid drugs; RAI: radioactive iodine

Characteristic	n (%)
Remission from ATDs	40 (62.5%)
ATD stopped upon remission	19 (50.0%)
Duration of ATD till remission in months	14.5 (10.3-22.8)
Total duration of ATD in months	22.5 (12.8-37.0)
Patient achieved remission with modality other than ATD	15/21 (71.4%)
Other modalities that achieved remission	
Radioactive iodine	11 (73.3%)
Surgery	4 (26.7%)
Relapsed patients	14/40 (35%)
Duration till relapse in months	12.0 (6.8-25.5)
Relapse happened while on ATD	6 (46.2%)
Treatment of relapse	
ATD	8 (61.5%)
RAI	5 (38.5%)
Surgery	2 (15.4%)

Factors associated with remission associated with ATD use

Table 3 presents the results of the factors associated with remission among the study participants. Among the demographic and clinical factors, gender showed a significant association with remission (p < 0.001), where only 50% of the females achieved remission compared to 94.4% of the males. Body mass index (BMI) at diagnosis also demonstrated significance (p = 0.028), with patients who achieved remission having a lower median BMI (26.4, IQR: 22.4-31.1) compared to those who did not (30.4, IQR: 26.2-36.7). Regarding laboratory parameters, the TSH level while on treatment exhibited significant association as the remitted group had higher maximum TSH levels (median 6.5) compared to the non-remitted group (median 1.2) (p = 0.010). Other variables like smoking history, goiter, presence of orbitopathy, thyroid autoantibodies, vitamin D, and calcium levels did not show significant associations with remission (Table 3).

**Table 3:** Patients’ characteristics in relation to remission Wilcoxon rank-sum test was used for numerical variables, and Fisher’s exact test or Pearson’s chi-squared test was used for categorical variables. A p-value <0.05 was considered statistically significant. Abbreviations: BMI: body mass index; ATD: anti-thyroid drugs; TSH: thyroid-stimulating hormone; PTH: parathyroid hormone; FT4: free T4; FT3: free T3, TRAb: TSH-receptor antibodies

Characteristic	Remission		p-value
	No N = 24	Yes N = 40	
Gender			<0.001
Male	1 (5.6%)	17 (94.4%)	
Female	23 (50%)	23 (50%)	
Age at diagnosis in years	41.0 (30.0-49.5)	36.5 (29.8-54.0)	0.983
BMI	30.4 (26.2-36.7)	26.4 (22.4-31.1)	0.028
Goiter	12 (63.2%)	21 (67.7%)	0.740
Volume/weight of thyroid gland in grams	25.6 (22.8-28.4)	49.0 (29.4-54.0)	0.381
Size of the right lobe of the thyroid gland in cm	4.8 (4.4-5.7)	5.1 (4.5-5.9)	0.565
Size of the left lobe of the thyroid gland in cm	4.5 (3.5-5.4)	4.9 (4.5-5.4)	0.108
Orbitopathy	3 (21.4%)	11 (78.6%)	0.492
Lab parameters at diagnosis (unit)			
TSH (mIU/L)			0.144
<0.01	21 (87.5%)	39 (97.5%)	
>=0.01	3 (12.5%)	1 (2.5%)	
FT4 (pmol/L)	26.8 (17.1-32.4)	29.5 (21.9-35.8)	0.178
FT3 (pmol/L)	11.9 (6.4-16.3)	9.2 (6.5-14.1)	0.712
TRAb level (IU/L)	7.1 (5.1-25.7)	6.7 (4.1-16.0)	0.668
TPO level (IU/mL)	1,970.3 (1,158.4-2,782.1)	114.5 (42.5-394.2)	0.154
Thyroglobulin-receptor antibodies (IU/mL)	47.9 (34.9-60.9)	39.2 (23.5-327.7)	0.923
Vitamin D (nmol/L)	54.4 (48.0-68.3)	35.2 (25.7-54.7)	0.132
Calcium level (mmol/L)	2.3 (2.2-2.4)	2.3 (2.2-2.4)	0.654
PTH level (pg/mL)	83.0 (50.7-116.5)	139.1 (116.1-147.0)	0.093
Degree of uptake on thyroid scan in %	3.8 (2.4-13.4)	6.3 (2.9-11.3)	0.846
Maximum TSH while on treatment (mIU/L)	1.2 (0.5-6.2)	6.5 (2.7-11.5)	0.010
Total duration of ATD in months	24.0 (9.0-36.0)	20.0 (14.0-41.0)	0.603

Side effects and hypothyroidism due to ATDs

In general, 10 patients (out of 46 available records) developed hypothyroidism after ATD treatment (21.7%). Furthermore, side effects occurred in 18 patients (28.1%). Neutropenia was the most common side effect (66.7%), followed by rashes (27.8%).

Characteristics of relapse

Among 40 patients with available records, relapse was reported among 14 patients (35.0%). Of them, relapse occurred within a median duration of 12.0 months (IQR = 6.8 to 25.5), and treatment of relapse was commonly by ATD (61.5%, Table 2). Based on the inferential analysis, relapse was not associated with patients’ characteristics, lab parameters, or ATD characteristics (Tables 4-6). Therefore, we could not construct a multivariable regression model to assess the predictors of relapse. 

**Table 4:** Patient characteristics in relation to relapse (categorical variables) Fisher’s exact test or Pearson’s chi-squared test was used for categorical variables. A p-value <0.05 was considered statistically significant.

Characteristic	Relapse		p-value
	No N = 26	Yes N = 14	
Gender			0.715
Male	8 (72.7%)	3 (27.3%)	
Female	18 (62.1%)	11 (37.9%)	
Smoking history	5 (29.4%)	2 (20.0%)	0.678
Goiter	17 (77.3%)	7 (70.0%)	0.681
Orbitopathy	10 (45.5%)	2 (22.2%)	0.418

**Table 5:** Patient characteristics in relation to relapse (numerical variables) Wilcoxon rank-sum test was used for numerical variables. A p-value <0.05 was considered statistically significant. Abbreviations: BMI: body mass index; ATD: anti-thyroid drugs

Characteristic		Relapse			p-value
		No N = 26	Yes N = 14		
Age at diagnosis in years	40.0 (30.3-48.8)		30.5 (26.5-51.0)	0.307	
BMI	30.5 (24.8-33.6)		27.2 (24.5-31.0)	0.604	
Size of the right lobe of the thyroid gland in cm	4.7 (4.3-5.5)		5.1 (4.4-5.7)	0.537	
Size of the left lobe of the thyroid gland in cm	4.5 (3.9-5.2)		5.0 (4.6-5.8)	0.063	
Degree of uptake on thyroid scan in %	5.6 (2.4-8.6)		8.6 (3.8-18.9)	0.059	
Total duration of ATD in months	19.0 (14.0-32.8)		24.0 (15.0-48.0)	0.395	

**Table 6:** : Laboratory parameters of patients in relation to relapse Wilcoxon rank-sum test was used for numerical variables, and Fisher’s exact test was used for categorical variables. A p-value <0.05 was considered statistically significant. Abbreviations: TSH: thyroid-stimulating hormone; FT4: free T4; FT3: free T3, TRAb: TSH-receptor antibodies

Characteristic	Relapse				p-value
	No N = 26		Yes N = 14		
Lab parameters at diagnosis (unit)					
TSH (mIU/L)				0.533	
<0.01		24 (92.3%)	14 (100.0%)		
>=0.01		2 (7.7%)	0 (0.0%)		
FT4 (pmol/L)		28.3 (21.8-31.3)	30.1 (23.8-43.1)	0.202	
FT3 (pmol/L)		9.4 (6.4-15.2)	9.3 (7.4-24.0)	0.371	
TRAb level (IU/L)		8.2 (3.7-27.7)	15.6 (10.1-16.0)	0.937	
Maximum TSH while on treatment (mIU/L)		6.9 (3.8-13.2)	5.3 (1.9-24.8)	0.377	

## Discussion

The study aimed to evaluate the remission rates, predictors of remission, relapse rates, and drug safety associated with ATD treatment in a population of patients with GD. The findings revealed a remission rate of 62.5%, which aligns with previous literature reports. Several factors were identified as predictors of remission, including gender, BMI, and maximum TSH levels. Male patients had a higher remission rate compared to females, while lower BMI levels were associated with a higher remission rate. Higher maximum TSH levels during treatment were also associated with achieving remission.

The present study documented an observed remission rate of 62.5%, which aligns with the range reported in previous literature [17]. Our investigation revealed a higher remission rate among males in comparison to their female counterparts. While concordant findings have been reported in prior studies, discrepant results have also surfaced. For instance, a recent investigation conducted in 2021 demonstrated a higher remission rate among females, whereas an earlier study from 2019 indicated a higher remission rate among males within the cohort treated with ATDs [22,23].

Furthermore, our study revealed an inverse correlation between BMI and remission rate, suggesting that lower BMI levels were associated with a heightened probability of remission achievement. Notably, scant literature discussing the influence of BMI on alterations in the remission rate was identified. In addition, elevated maximum TSH levels demonstrated an inverse relationship with the remission rate. This finding aligns with the conclusions drawn by Young Kwang Choo et al., whose investigation showcased that elevated TSH levels during MMI administration constituted a favorable prognostic indicator for remission [24]. Moreover, another study highlighted a positive association between higher TSH levels during treatment and the duration until remission [25].

Regarding the duration until remission, our study indicated an average of 14.5 months, with no statistically significant associations revealed between the remission rate and the duration of ATD use. Nonetheless, it is worth noting that diverse research endeavors have yielded disparate outcomes in this regard. Several investigations propose that long-term therapy (exceeding 60 months) confers superior benefits compared to conventional therapy (ranging from 12 to 24 months), as evidenced by higher remission rates and reduced relapse rates [26,27].

Among the available records of 40 patients, a total of 14 patients (35.0%) experienced a relapse, which is consistent with previous research findings indicating relapse rates ranging from 30% to 40% [17]. The relapse events typically occurred within a median duration of 12.0 months, with an IQR spanning from 6.8 to 25.5 months. The predominant form of treatment administered to the patients was ATDs, accounting for 61.5% of the cases (refer to Table 2). This was followed by radioactive iodine (RAI) treatment in 38.5% of the cases and surgery in 15.4% of the cases.

To the best of our knowledge, previous studies have identified certain factors that potentially serve as predictors or indicators of the likelihood of relapse. These factors include younger age, larger thyroid volume, greater goiter size, and elevated levels of T3 and T4 hormones [28]. However, after conducting inferential analysis, it was determined that relapse did not exhibit any significant associations in our study with the patients' characteristics, laboratory parameters, or ATD characteristics. Consequently, it was not feasible to develop a multivariable regression model to identify predictors of relapse. This could be because of the relatively small sample size in our study and some missing data.

In line with other pharmacological agents, the administration of ATDs is not devoid of potential risks or side effects. Adverse events associated with ATDs can be categorized into two distinct groups: major events, characterized by severe outcomes such as agranulocytosis, hepatotoxicity, pancreatitis, or vasculitis, and minor events, encompassing less severe manifestations such as rash, gastric intolerance, and arthralgia. Within the scope of our study, a subset of 18 patients (comprising 28.1% of the total cases) reported experiencing side effects attributable to ATD therapy. Among the documented side effects, neutropenia emerged as the most prevalent, accounting for 66.7% of the reported cases, followed by rash at 27.8%. Notably, we observed that hypothyroidism developed in 10 out of the 46 patients for whom comprehensive records were available subsequent to ATD treatment, representing a prevalence rate of 21.7%. It is noteworthy, however, that existing literature reports a lower incidence of hypothyroidism at 10% [29]. This hypothyroidism is most likely related to ATD and is transient and would resolve after decreasing or stopping the agent.

The predominance of MMI as the primary ATD in our study, accounting for 98.4% of cases with a median dose of 10.0 mg/day, may have influenced the observed remission rates. This preference for MMI could be attributed to its availability, cost-effectiveness, and strong clinical recommendations. Moreover, a study comparing MMI with PTU found that a daily dose of 15 mg of MMI was significantly more effective in inducing euthyroidism than a much higher dose of 150 mg PTU, suggesting that MMI is the preferred choice for once-daily antithyroid treatment [30]. This preference for MMI is likely to have contributed to the favorable remission outcomes observed in our patient cohort.

The current study has several limitations that should be considered. First, the sample size of the study was relatively small, with only 64 participants. This limited sample size may restrict the generalizability of the findings and reduce the statistical power to detect subtle associations or predictors. Second, the research was conducted exclusively at a single institution, which introduces the potential for institutional biases and restricts the diversity of the patient population. As a result, the results may not fully represent the broader population of GD patients. Third, the study relied on retrospective analysis of existing medical records, which carries the inherent risk of incomplete or inaccurate documentation. This may introduce information bias and affect the reliability of the data, potentially leading to biased results. In addition, there were missing data points for some variables, which may introduce bias and compromise the completeness of the analysis. Thus, the absence of these data points and the small sample size reduced the precision and generalizability of the findings. This also prevented identifying relapse predictors, leaving a gap in understanding long-term outcomes. Lastly, our multivariable analysis did not account for all potentially relevant factors, such as treatment adherence or genetic predispositions, which further limits the comprehensiveness of our findings. More research is needed to address these gaps, as it could help identify key relapse predictors and improve the understanding of long-term outcomes, ultimately guiding more effective treatment strategies.

## Conclusions

Overall, the findings highlight the predominance of MMI as the primary ATD used and the favorable remission outcomes observed in the majority of patients. Factors such as male gender, lower BMI, and maximum TSH levels, were identified as predictors of remission. Further research with larger sample sizes, multi-center collaboration, and longer follow-up periods is warranted to validate these findings and enhance our understanding of the management of GD.
